# Therapist-Supported Internet-Based Cognitive Behavior Therapy for Stress, Anxiety, and Depressive Symptoms Among Postpartum Women: A Systematic Review and Meta-Analysis

**DOI:** 10.2196/jmir.6712

**Published:** 2017-04-28

**Authors:** Ying Lau, Tha Pyai Htun, Suei Nee Wong, Wai San Wilson Tam, Piyanee Klainin-Yobas

**Affiliations:** ^1^ Alice Lee Centre for Nursing Studies Yong Loo Lin School of Medicine National University of Singapore Singapore Singapore; ^2^ Medical Resource Team, National University of Singapore Libraries Singapore Singapore

**Keywords:** Internet, post-traumatic stress disorders, stress, anxiety, depression, cognitive behavior therapy, meta-analysis

## Abstract

**Background:**

A growing number of meta-analyses have supported the application of therapist-supported Internet-based cognitive behavior therapy (iCBT) for psychological disorders across different populations, but relatively few meta-analyses have concentrated on postpartum women.

**Objective:**

This meta-analysis evaluated the efficacy of therapist-supported iCBT in improving stress, anxiety, and depressive symptoms among postpartum women.

**Methods:**

A total of 10 electronic databases were used to search for published and unpublished trials. Cochrane Collaboration tool for assessing risk of bias was utilized to measure methodological quality. Meta-analysis was performed using the RevMan software (Review Manager version 5.3 for Windows from the Nordic Cochrane Centre, the Cochrane Collaboration, 2014). Among the 789 studies identified, 8 randomized controlled trials were selected, involving 1523 participants across 6 countries.

**Results:**

More than half (65%) of the eligible studies had a low risk of bias with no heterogeneity. Results revealed that therapist-supported iCBT significantly improved stress (*d*=0.84, n=5), anxiety (*d*=0.36, n=6), and depressive symptoms (*d*=0.63, n=8) of the intervention group compared with those of the control group at post-intervention.

**Conclusions:**

This review revealed that therapist-supported iCBT significantly improves stress, anxiety, and depressive symptoms among postpartum women with small to large effects. Future effectiveness studies should establish the essential components, format, and approach of iCBT with optimal levels of human support to maximize a long-term effect.

## Introduction

Cognitive behavior therapy (CBT) is a form of psychotherapy based on the assumption that all psychological disturbances are caused by dysfunctional thinking [1]. CBT aims to modify thoughts, beliefs, and perceptions, and change behavioral pattern [2]. Cognitive restructuring is a common psychotherapeutic process in CBT for identifying, evaluating, and changing negative, distorted thoughts and beliefs [3]. Cognitive restructuring is a useful technique to understand wrong automatic beliefs and it helps individuals to reframe their negative perception or distorted thinking in a more positive frame of mind [3]. Behavioral activation, which is a different form of CBT, is a functional analytical approach for engaging in enjoyable activities frequently to maintain or improve psychological well-being [4]. Behavioral activation is a development of activity scheduling that focuses on the use of avoided activities as a guide for activity scheduling and functional analysis of cognitive processes that involve avoidance [4]. Despite the well-established efficacy of CBT for treating and preventing psychological disorders [5], barriers to the administration of CBT exist, which may include insufficient therapists, stigmatization, geographical remoteness, long waiting times, and high costs [6]. CBT has been suggested suitable for remote delivery because of its structured content [7]. During the past 15 years, the development of Internet-based psychological intervention has progressed significantly [8], particularly in light of the rapid improvements in Internet technologies globally [9]. An innovative administration has been developed in the form of Internet-based CBT (iCBT) to minimize treatment barriers and increase access to care [8,10].

The Internet makes CBT feasible and worth consideration. Implementation of iCBT is able to administer the full course of a CBT treatment using online self-help format, which might or might not be supported by a therapist [8]. A therapist can provide support through phone, email, text, or an interactive computer interface [10,11]. Therapist-supported iCBT is a therapy that is guided by an identified therapist who gives feedback and answers to questions, and which can include interactive features through the Internet to get access to psychological treatment [8]. Recent systematic reviews of therapist-supported iCBT for anxiety [10] and psychiatric and somatic disorder [12] produced equivalent effect compared with face-to-face CBT. Therapist-supported interventions involving higher levels of human support improve outcomes for depression, but do not significantly affect outcomes for stress [13] or anxiety [10]. An Internet-based intervention with instant feedback has achieved adherence and effectiveness similar to that of the same intervention with human support [14]. The potential benefits of therapist-supported iCBT are customizability, cost-effectiveness, time-effectiveness, geographic flexibility, time flexibility, consistency, high availability, and rapid dissemination [15,16].

The postpartum period is a highly challenging time for women because of changes in physical, familial, financial, occupational, and other realms [17]. Changes may affect a woman’s psychosocial and physical resources, resulting in stress, anxiety, and depressive symptoms [17,18]. Reported prevalence rates suggest that 14.3% of women suffer from general stress [18], 1%-30% from post-traumatic stress symptoms [19], 24.9% from anxiety [20], and 13%-19% from depressive symptoms [21] during the postpartum period. In addition, evidence indicates that the coexistence of stress, anxiety, and depressive symptoms occur during the postpartum period [17]. Timely and efficacious intervention is important during the postpartum period, especially when considering the adverse short- and long-term maternal health outcomes and child development outcomes [22-24]. Strong evidence supports that CBT is effective for treatment and prevention during the postpartum period [25]. A review revealed that interventions initiated during the postpartum period were more effective than those initiated during the antenatal period and that the one-to-one therapy was more effective than the group therapy [25]. Notably, the Internet was found to be the preferred source of information among these women [20]. For example, 90% used the Internet to search for health-related information [20] and 69% used the Internet to seek formation about postpartum depression [26]. They expressed interest in use of Web-based resources and greater engagement in ehealth behaviors related to mental health [27]. An increasing number of randomized controlled trials (RCTs) found that iCBT is effective for stress [28,29], anxiety [30,31], and depressive symptoms [32,33] among postpartum women. Given the burgeoning development of iCBT for a broad range of conditions [8] paralleled by the rapid increase in access to instant cyber connectivity, what is the effect of therapist-supported iCBT in improving stress, anxiety, and depressive symptoms among postpartum women?

Meta-analyses are applied in relation to a wide range of study designs; those that address trial designs are considered to represent the strongest evidence, and they have become increasingly prevalent over the years [34]. A growing number of meta-analyses have supported the application of therapist-supported iCBT for stress [13,35], anxiety [7,10], and/or depressive symptoms [36,37] across different populations, but relatively few meta-analyses of therapist-supported iCBT have concentrated on the postpartum population. We are aware of two recent systematic reviews [38,39] that investigate computer- or Web-based interventions for the prevention and treatment of perinatal mental health [38] or mood disorders [39]. The Two reviews suggest that computer- or Web-based intervention is effective for depressive symptoms [38,39], but mixed results were found for stress and anxiety symptoms [38]. However, the two reviews have been limited in using heterogeneous study designs [38,39], different therapeutic approaches [38], nonspecific outcomes [38,39], and a few eligible studies (n=4) [39]. Although one review [38] used a meta-analytical approach, their population was varied with antenatal, postpartum women, and their partners.

Evidently, therapist-supported iCBT provides an efficacious, accessible, and economically sound intervention for a broad range of conditions [8] across different populations [7,13]. A systematic review of existing evidence is necessary to determine whether therapist-supported iCBT for postpartum women is efficacious for prevention and treatment of stress, anxiety, and depressive symptoms before therapist-supported iCBT is embedded in routine clinical practice [39]. Further exploration is recommended to address the gaps in the current literature. The findings of this review could guide future studies exploring the next steps of therapist-supported iCBT implementation among the postpartum population. By conducting a systematic review and meta-analysis, we synthesized the best available evidence. This review aimed to systematically assess studies that examined therapist-supported iCBT interventions for improving stress, anxiety, and depressive symptoms among postpartum women.

## Methods

This review was conducted according to the standards outlined in the Preferred Reporting Items for Systematic Reviews and Meta-Analyses (PRISMA) statement [40]. The protocol is registered in the PROSPERO database (CRD42016039094).

### Eligibility Criteria

Studies were selected for the meta-analysis if they fulfilled the PICOS (population, intervention, comparison, outcomes and study) criteria [19]:

- Population: target women with age ≥ 18 years in the postpartum period (≤ 2 years postpartum) [41]
- Intervention: Therapist-supported iCBT was delivered over the Internet through the use of websites, email, phone, or Skype. The iCBT must have included support of therapists through phone, email, text, and an interactive computer interface. The intervention comprised at least one of the elements of CBT, including cognitive restructuring, behavioral activation, or skills training
- Comparison: attention control, waitlist, or treatment as usual (TAU)
- Outcomes: stress, anxiety, and depressive symptoms at postintervention
- Type of studies: RCTs

We excluded studies on teenage pregnancy (< 18 years). We did not include studies that had no CBT component in the intervention group; had active treatment containing a CBT component in the control group; were clinical controlled trials, cross-sectional, cohort, one-group pre- and posttest, and qualitative designs; only had abstracts and were study protocols, reviews, or conference papers.

### Search Strategy

Similar systematic review papers were searched from the Cochrane Databases of Systematic Reviews, Joanne Briggs Institute, Centre for Reviews and Dissemination, University of York, PubMed Clinical Queries, Google, and Google Scholar to verify that the papers had not been conducted recently. The search strategy aimed to find published or unpublished studies without time limitation to maximize the search. We did not restrict our search to studies reported in any particular language. However, we conducted searches in English.

A 3-step search strategy was employed from inception until February 9, 2017. The first phase was a comprehensive search using identified keywords and index terms, searching the following 10 electronic databases: EMBASE, PubMed, Cumulative Index to Nursing and Allied Health Literature, Academic Search Completed, PsycINFO, PsycARTICLES, Cochrane Library, Web of Science, Scopus, and ProQuest Dissertations and Theses. Index and keyword terms were used (Multimedia Appendix 1). The index terms and keywords were combined and truncated according to the syntax rules of each database.

The second phase involved searching ClinicalTrials.gov, WHO International Clinical Trials Registry Platform, and International Standard Randomised Controlled Trial for unpublished trials relevant to the review. When eligible trials were found, unpublished data were requested.

The third phase involved searching the reference lists of the included studies and checking previous reviews relevant to the topic for additional studies. The bibliographical software package EndNote program version X7 (Thomas Reuters) was used to import all the references and remove duplicates. The remaining studies were assessed independently against the inclusion and exclusion criteria by two authors (ie, LY and TP).

### Quality Assessment

After identifying full-text articles that fulfilled the selection criteria, the studies were submitted for quality assessment and verified for eligibility. Cochrane Collaboration tool for assessing risk of bias was used by the two authors to independently evaluate the potential for bias in each study [42]. The following indicators of internal validity specific to the methodology were collected: (1) random sequences generation, (2) allocation concealment, (3) blinding of participants and personnel, (4) blinding of outcome assessment, (5) incomplete outcome data, and (6) selective reporting [42]. Assessment related to the risk of bias was assigned with a judgment of “low risk” of bias, “high risk” of bias, or “unclear risk” of bias. Any difference in opinion between the two authors was resolved by consensus.

### Data Extraction

The characteristics of trials and elements of therapist-supported iCBT were extracted from each study through structured summaries. Items extracted for the characteristics of trials included authors, year, countries, design, sample with health condition, age, name of iCBT, control group, sample size, outcomes, attrition rate, follow-up, intention-to-Treat (ITT) analysis, and grant support. Items extracted for the descriptions of iCBT were: aim, numbers of sessions, components, therapy, support, provider, peer support, partner support, contact with therapist, communication, interactivity, activities, multimedia, and duration. The summary tables were thoroughly reviewed for accuracy and relevance by the two authors independently. Study authors were contacted for any missing or additional information.

### Data Analysis

Data were synthesized using the RevMan software (Review Manager Version 5.3 for Windows from the Nordic Cochrane Centre, the Cochrane Collaboration, 2014). We used the generic inverse variance method in our meta-analysis to combine the continuous outcomes with means and standard deviations (SD) [42]. The weight given to each trial was chosen to be the inverse of the variance of the effect estimate (ie, one over the square of its standard error) [42]. With the inverse variance method, mean difference was used for the scores of stress, anxiety, and depressive symptoms. The standardized mean differences with their corresponding 95% CI were employed to combine studies that measured the same outcomes with different methods. The test of the overall effect was assessed using Z-statistics at *P*<.05. To quantify the efficacy of therapist-supported iCBT on stress, anxiety, and depressive symptoms, we calculated the effect sizes by subtracting the mean value of the iCBT group from the mean value of the control group at posttest and dividing the result by the pooled SD of the two groups. The effect sizes were expressed as Cohen *d* or standardized mean difference, which were interpreted as small (0.2< *d*<0.5), medium (0.5< *d*<0.8), and large (*d* ≥0.8) [43].

Heterogeneity between studies was evaluated using the Cochrane Q (chi-square test) and *I*^2^ statistics in the RevMan software. The statistical significance for heterogeneity was set as *P*>.10, and estimates of the degree of heterogeneity using *I*^2^ were made by setting 0%–40% as might not be important, 30%–60% as moderate, 50%–90% as substantial, and 75%–100% as considerable [42]. We used fixed- and random-effects models in our meta-analysis according to heterogeneity between studies on different statistical assumptions [44]. The fixed-effects model was used to estimate one true effect in cases without significant heterogeneity (*P*<.10) because we assumed that the true effect size was the same in the eligible trials, whereas the random-effects model (tau-square) was employed to estimate the mean of a distribution of effects in cases with heterogeneity between studies (*P*>.10) and in those with *I*^2^ values of over 40% because we assumed that the true effect size varied from trial to trial [42]. Subgroup analysis was performed to (1) explore the source of heterogeneity and (2) evaluate the effect in a specific subgroup; performing a subgroup analysis provided information about essential elements for maximizing effectiveness of therapist-supported iCBT [45]. The predefined subgroups differed in health condition, control conditions, type of iCBT, number of sessions, and professional support. We planned to construct funnel plots to determine the possible influence of publication biases if the number of eligible trials was more than 10 [42].

## Results

### Study Selection

**Figure 1:**
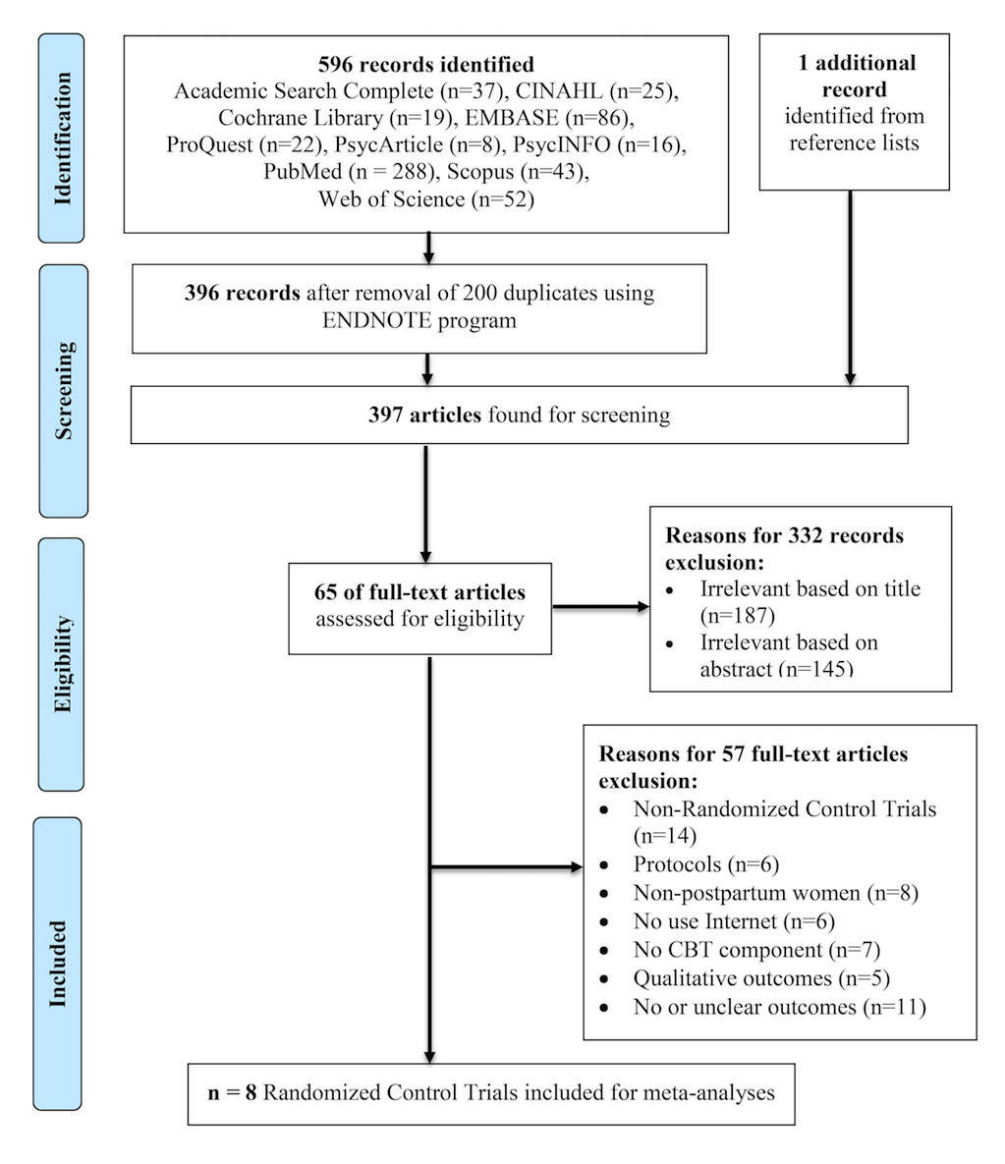
Preferred reporting items for systematic reviews and meta-analysis (PRISMA) diagram displaying procedure for trials selection.

The search and selection of articles is illustrated in Figure 1. Using the specified search terms, the searched 10 databases produced a total of 596 records. Among these studies, 200 article duplicates were removed. One additional record was identified from the reference list. On the basis of an analysis of the words in paper titles and abstracts, 332 records were excluded. Full-text articles of the remaining 65 articles were retrieved, reviewed, and selected on the basis of relevance and quality for eligibility. A total of 57 studies were excluded for reasons outlined in Figure 1. Finally, 8 RCTs were selected for meta-analysis.

### Risk of Bias in Included RCTs

**Figure 2:**
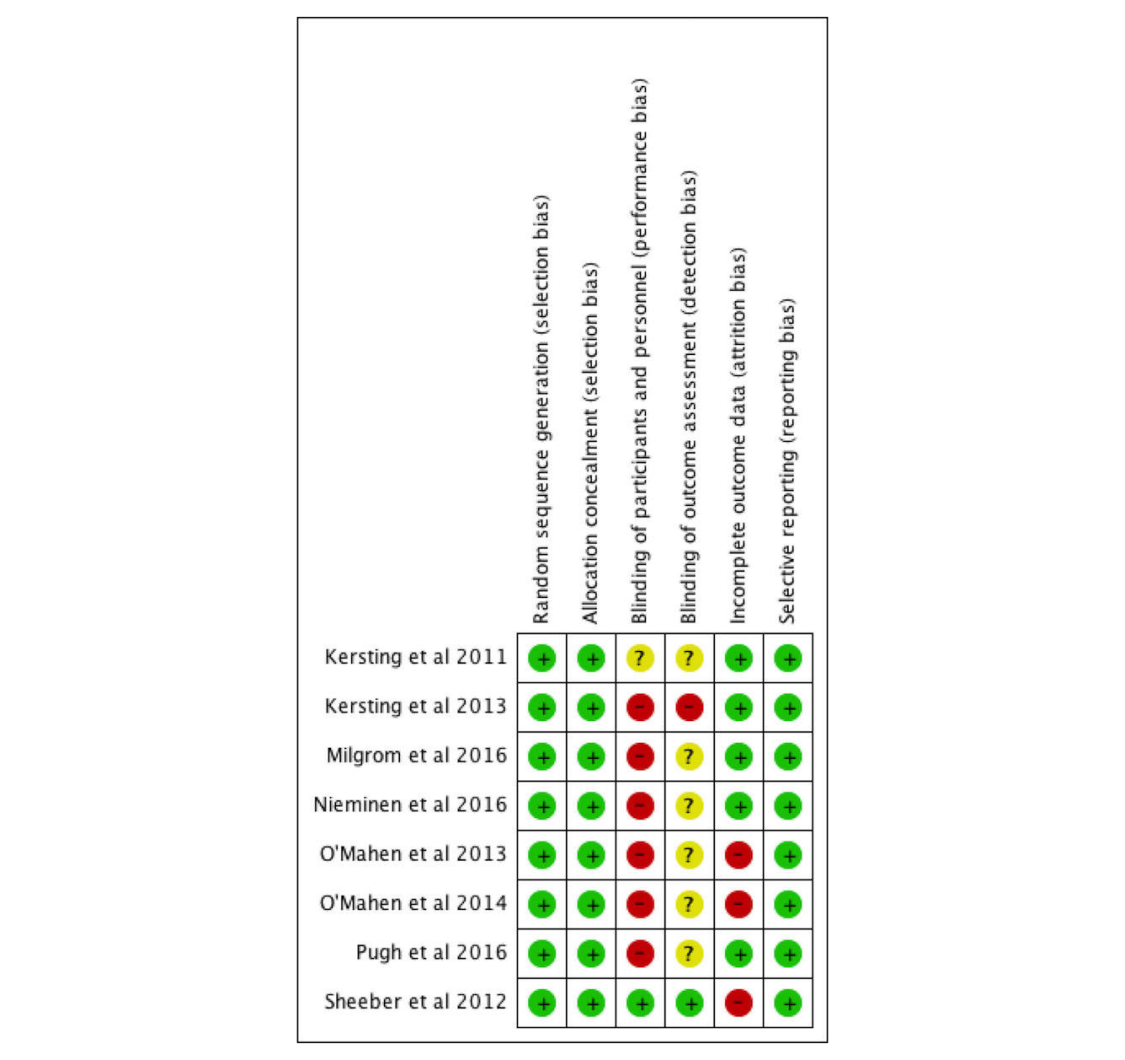
Risk of bias summary. These are authors’ judgments of each methodological quality item for each included study. Plus signs (+) indicate high methodological quality (low risk of bias); minus signs (-) indicate low methodological quality (high risk of bias); question marks (?) indicate unclear methodological quality (reported information about what happened in the study was insufficient).

We summarized the findings for the risk of bias graph and summary in Figure 2 and Multimedia Appendix 2, respectively. In all, 8 studies (100%) had adequate sequence generation for randomization. All of them (100%) had adequate allocation concealment. Only one study (13%) had implemented the blinding of participants and personnel and outcome assessment. Whereas 6 trials (75%) were unclear for the blinding of outcome assessment, 1 trial (13%) was unclear to implement the blinding of participants and personnel. Of the 8 studies, 5 (63%) addressed low risk of bias concerning incomplete outcome data. All of the studies (100%) had low risk of bias for selective reporting. The two authors independently checked for risk of bias. The interrater agreement was 100% for global ratings, with two minor disagreements at the component level, but these disagreements were resolved through discussion. The number of trials (n<10) in this review was insufficient to make a meaningful funnel plot to determine publication bias because the test power was excessively low to distinguish chance from real funnel plot asymmetry [42].

### Characteristics of Included Studies

This meta-analysis included 8 studies [28-33,46,47] with a total of 1523 participants conducted across 6 countries (Table 1), including Australia (13%, 1/8) [28], Canada (13%, 1/8) [31], Germany (25%, 2/8) [46,47], Sweden (13%, 1/8), the United Kingdom (25%, 2/8) [30,32], and the United States (13%, 1/8) [33]. All of the studies were conducted between 2011 [47] and 2016 [31], with 2016 having the highest number of publications (38%, 3/8) [28,29,31].

The mean age of the participants in the studies ranged from 31 [33] to 35 years [29]. Participants were postpartum women with 3 different health conditions, namely, depressive symptoms (63%, 5/8) [28,30-33], post-traumatic stress symptoms (13%, 1/8) [29], and pregnancy loss (25%, 2/8) [46,47]. The sample sizes were between 43 [28] and 910 [32]. Comparators were waitlist treatment (50%, 4/8) [29,31,46,47], usual care (38%, 3/8) [28,30,32], and waitlist or usual care (13%, 1/8) [33]. Six of the studies (75%, 6/8) assessed more than one target outcome. Whereas the rest did not report any follow-up, 6 of the studies had a follow-up for 1 [31] to 12 months [46] after intervention. Attrition rates ranged from 3% [33] to 60.8% [32] and from 0% [33] to 63.8% [32] for the intervention and control groups, respectively. Of the all the selected articles, 5 (63%, 5/8) used ITT analysis, and majority of them (88%, 7/8) were supported by grants.

**Table 1:** Characteristics of the eligible 8 randomized controlled trials.

Author (year)	Country	Health condition (recruitment)	Age (in years) (mean)	iCBT^a^ (I)	Control (C)	Sample size (N)	Outcomes (measures)	Follow-up (in months)
Kersting et al (2011)^b,c^ [47]	Germany	Pregnancy loss (–)	>18 (34)	Manualized CBT^d^ treatment program	Waitlist	I: 48 C: 35	Stress (IES)^e^ Anxiety (BSI-GSI)^f^ Depression (BSI-GSI)^f^	3
Kersting et al (2013)^b,c^ [46]	Germany	Pregnancy loss (–)	>18 (34)	Internet-based intervention for parents after parental loss	Waitlist	I: 115 C: 113	Stress (IES-R)^g^ Anxiety (BSI-GSI)^f^ Depression (BSI-GSI)^f^	3, 12
Milgrom et al (2016)^b,c^ [28]	Australia	Major or minor depression (< 1 year postpartum)	>18 (32)	MumMood Booster	TAU^h^	I: 21 C: 22	Stress (DASS-21)^i^ Anxiety (DASS-21)^i^ Depression (BDI-II)^j^	3
Nieminen et al (2016)^b,c^ [29]	Sweden	Post-traumatic stress (> 3 months postpartum)	>18 (35)	Internet-based trauma-focused CBT^d^	Waitlist	I: 28 C: 28	Stress (IES-R)^g^ Anxiety (BAI)^k^ Depression (BDI-II)^j^	(–)
O’Mahen et al (2013)^c^ [32]	United Kingdom	Depressive symptoms (< 12 months postpartum)	>18 (32)	Postnatal internet-based behavioral activation	TAU^h^	I: 462 C: 448	Depression (EPDS)^l^	(–)
O’Mahen et al (2014)^c^ [30]	United Kingdom	Depressive symptoms (< 12 months postpartum)	>18 (–)	Netmums Helping with Depression	TAU^h^	I: 41 C: 42	Anxiety (GAD-7)^m^ Depression (EPDS)^l^	6
Pugh et al (2016)^b^ [31]	Canada	Depressive symptoms (< 12 months postpartum)	>18 (–)	Therapist-assisted iCBT^a^	Waitlist	I: 25 C: 25	Stress (DASS-21)^i^ Anxiety (DASS-21)^i^ Depression (EPDS)^l^	1
Sheeber et al (2012)^c^ [33]	United States	Depressive symptoms (–)	>18 (31)	Mom-Net intervention	Waitlist or TAU^h^	I: 35 C: 35	Depression (BDI-II)^j^	3

^a^iCBT: Internet-based cognitive behavioral therapy.

^b^These studies used intention-to-treat analysis.

^c^These studies had grant support.

^d^CBT: cognitive behavioral therapy.

^e^IES: Impact of Event Scale.

^f^BSI-GSI: Brief Symptom Inventory-Global Severity Index.

^g^IES-R: Impact of Event Scale-revised.

^h^TAU: treatment as usual.

^i^DASS-21: Depression Anxiety Stress Scale.

^j^BDI-II: Beck Depression Inventory-II.

^k^BAI: Beck Anxiety Inventory.

^l^EPDS: Edinburgh Postnatal Depression Scale.

^m^GAD-7: Generalized Anxiety Disorder Scale.

### Descriptions of Internet-Based Cognitive Behavior Therapy

Detailed descriptions of iCBT are presented in Multimedia Appendices 3 and 4. All iCBTs were based on cognitive-behavioral strategies, including cognitive restructuring [29,46,47] and behavioral activation [30,33] with additional elements of psychoeducation [31], parenting preparation, or parenting focus [33]. The numbers of sessions ranged from 6 [47] to 12 [30]. All therapies used one-to-one setting. Therapists support all therapies and 3 of them incorporated a self-monitoring or self-help component [28,30,33]. All of the studies had human support coming from psychologists or clinical psychologists (75%, 6/8) [28,29,31,32,46,47], master’s or PhD psychology students (25%, 2/8) [29,31], CBT-trained mental health workers (25%, 2/8) [32,33], and a layperson (13%, 1/8) [33].

Peer support using chat room discussion was used by 4 of the therapies [28,30,32,33], and only 1 trial used partner support using websites [28]. Majority of the therapies were asynchronous (88%, 7/8) [29-33,46,47] and 1 was synchronous [28] in terms of two-way feedback communication with therapists using websites (88%, 7/8) [28-30,32,33,46,47], phone calls (63%, 5/8) [28,30-33], emails (38%, 3/8) [30-32], and text messages (13%, 1/8) [30]. The delivery modes included multimedia and interactive formats, such as animation, video, audio, or photos [28,31]. The activities of the therapies included text-based reading (63%, 5/8) [28,29,31,46,47], assignment or homework (88%, 7/8) [28-32,46,47], interactive exercise (13%, 1/8) [30], expressive writing (38%, 3/8) [33,46,47], and online clinics or consultation (25%, 2/8) [32,33]. The duration of the therapies varied among the 8 studies, ranging from 5 [46,47] to 17 weeks [30].

### Efficacy of Therapist-Supported iCBT on Stress Symptoms

**Figure 3:**
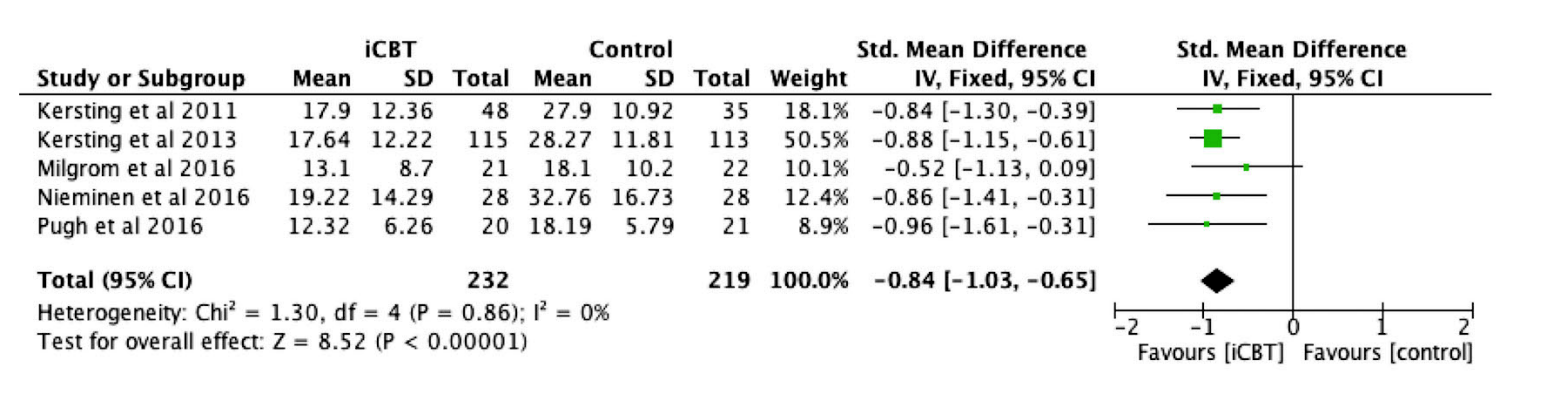
Forest plot of standardized mean difference (95% CI) in change of stress symptoms scores for Internet-based cognitive behavior therapy intervention and control group.

By comparing the intervention and control groups using stress symptoms at post-intervention as a dependent variable, 5 studies [28,29,31,46,47] assessed the efficacy of therapist-supported iCBT among 451 women. Depression Anxiety Stress Scale-21 (DASS-21) [48], Impact of Event Scale (IES) [49] and IES-Revised [50] were used to measure stress symptoms. Figure 3 shows that therapist-supported iCBT interventions had a large effect size of .84 (95% CI 0.65-1.03) on eliminating stress symptoms. The overall effect (Z=8.52, *P*<.001) was significant, but *I*^2^ showed 0%, and the *P* value of chi-square was .86. A series of subgroup analyses was performed to explore more information about the therapist-supported iCBT intervention, as shown in Table 2. Subgroup differences were found insignificant for stress symptoms based on health condition, control condition, number of sessions, peer support, and professional support. Interestingly, we observed the therapist-supported iCBT with waitlist as comparator and exclusive therapist support were significant (*d*=0.88, Z=8.43, *P*<.001) on eliminating stress symptoms, whereas intervention using TAU as comparator and therapist support with self-help component did not (*d*=0.52, Z=1.66, *P*>.05). However, only 1 study in the subgroups had limited generalizability and thus further trials were warranted.

### Efficacy of Therapist-Supported iCBT on Anxiety Symptoms

**Figure 4:**
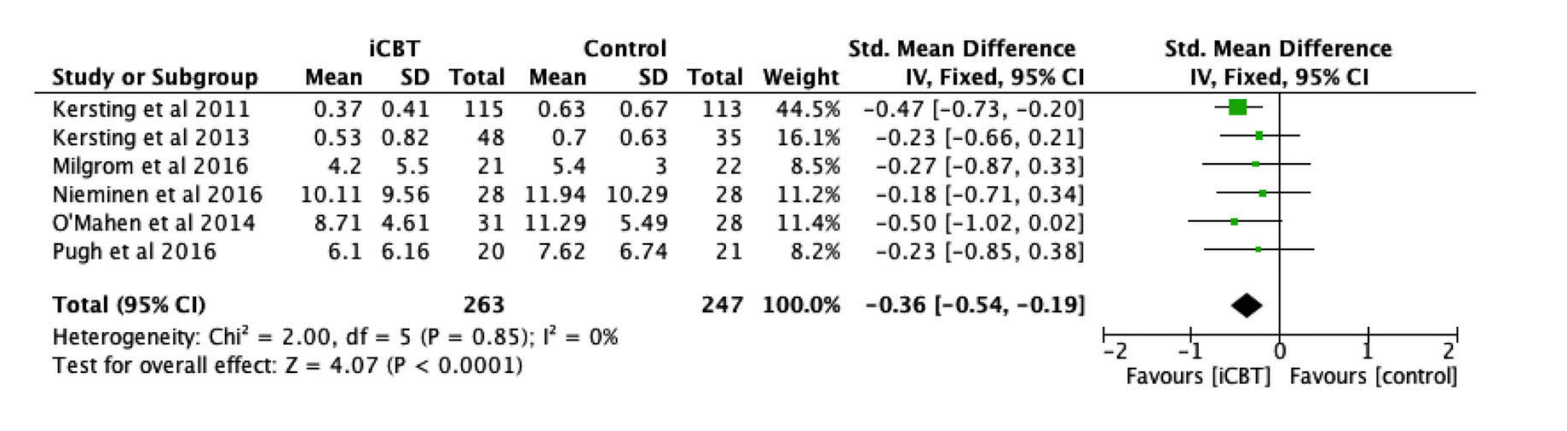
Forest plot of standardized mean difference (95% CI) in change of anxiety symptoms scores for Internet-based cognitive behavior therapy intervention and control group.

Using anxiety symptoms at post-intervention as a dependent variable in 6 studies [28-31,46,47], the efficacy of therapist-supported iCBT was evaluated among 510 participants by comparing the intervention and control groups. Anxiety symptoms were measured through the Brief Symptom Inventory-Global Severity Index (BSI-GSI) [51], DASS-21 [48], Generalized Anxiety Disorder Scale [52], and Beck Anxiety Inventory [53]. Therapist-supported iCBT exerted a significant effect on improving anxiety symptoms (Z=4.07, *P*<.001) with small to medium effect size (*d=* 0.36), as shown in Figure 4. Meta-analysis on anxiety symptoms showed no heterogeneity (*I*^2^=0%). Significant subgroup differences were not found for anxiety symptoms based on health condition, control condition, type of iCBT, number of sessions, and professional support (Table 2).

### Efficacy of Therapist-Supported iCBT on Depressive Symptoms

**Figure 5:**
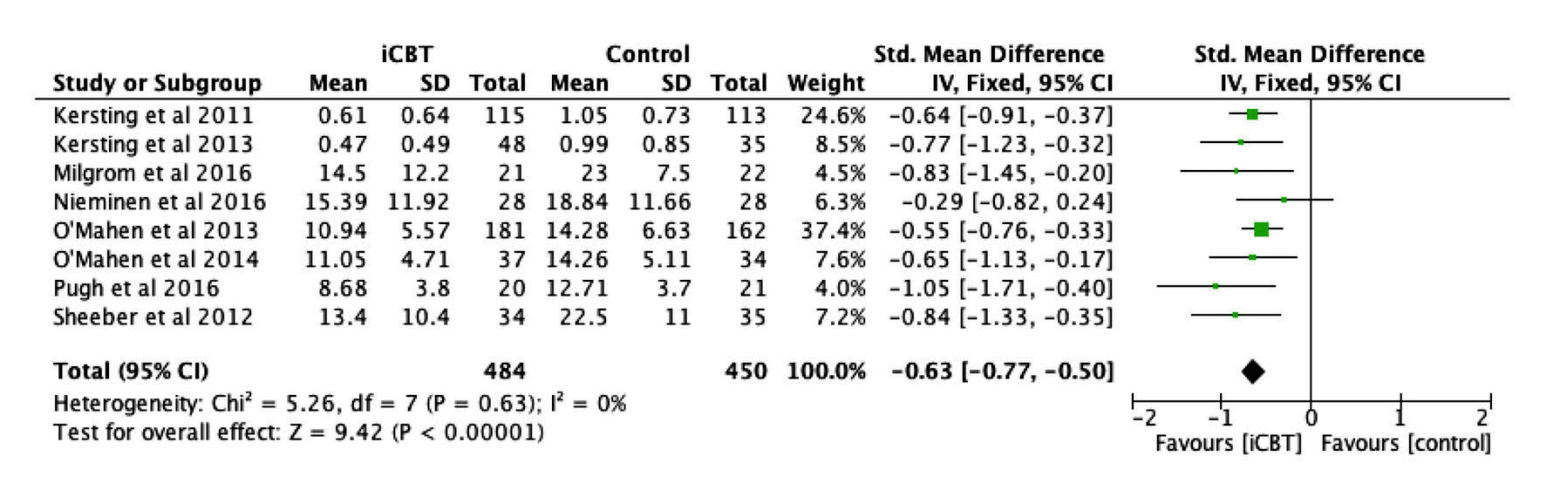
Forest plot of standardized mean difference (95% CI) in change of depressive symptoms scores for Internet-based cognitive behavior therapy intervention and control group.

A total of 8 studies [28-33,46,47] assessed the efficacy of therapist-supported iCBT interventions among 934 participants by comparing the intervention and control groups using depressive symptoms at post-intervention as dependent variable. Depressive symptoms were measured through the Beck Depression Inventory-II [54], Edinburgh Postnatal Depression Scale [55], and BSI-GSI [51]. iCBT interventions in this meta-analysis exerted a significant effect on improving depressive symptoms (Z=9.42, *P*<.001) with medium to large effect size of 0.63 (Figure 5). The meta-analysis of these 8 studies showed no heterogeneity (*I*^2^=0%). No significant subgroup differences were found for depressive symptoms according to health condition, control condition, type of iCBT, number of sessions, and professional support (Table 2).

**Table 2:** Subgroup analyses of Internet-based cognitive behavioral therapy (iCBT) on stress, anxiety, and depressive symptoms.

Subgroup analyses			Number of comparisons	Effect size, *d*	95% CI	Z-statistics	*P*	Heterogeneity, *I*^2^ (%)	Chi-square, *χ*^2^ (*df*), comparison
Stress symptoms [28,29,31,46,47]									
	**Health condition**								
		Depression [28,31]	2	0.72	0.28-1.17	3.18	.001	0	0.3 (2)
		Pregnancy loss [46,47]	2	0.87	0.64-1.10	7.31	<.001	0	
		Post-traumatic stress [29]	1	0.84	0.65-1.03	3.06	.002	N/A	
	**Control condition**								
		Waitlist [29,31,46,47]	4	0.88	0.64-1.08	8.43	<.001	0	1.2 (1)
		Treatment as usual [28]	1	0.52	0.09-1.13	1.66	.10	N/A	
	**Number of sessions**								
		< 8 sessions [28,31,46,47]	4	0.84	0.63-1.05	7.95	<.001	0	0.0 (1)
		≥ 8 sessions [29]	1	0.86	0.31-1.41	3.06	.002	N/A	
	**Professional support**								
		Exclusive therapist support [29,31,46,47]	4	0.88	0.67-1.08	8.43	<.001	0	1.2 (1)
		With self-help component [28]	1	0.52	0.09-1.13	1.66	.10	N/A	
Anxiety symptoms [28-31,46,47]									
	**Health condition**								
		Depression [28,30,31]	3	0.35	0.02-0.68	2.09	.04	0	0.6 (2)
		Pregnancy loss [46,47]	2	0.40	0.18-0.63	3.50	<.001	0	
		Post-traumatic stress [29]	1	0.18	0.34-0.71	0.68	.50	N/A	
	**Control condition**								
		Waitlist [29,31,46,47]	4	0.35	0.16-0.55	3.54	<.001	0	0.1 (1)
		Treatment as usual [28,30]	2	0.40	0.01-0.80	2.01	.04	0	
	**Type of iCBT^a^**								
		Behavioral activation [30]	1	0.50	0.02-1.02	1.90	.06	N/A	0.3 (1)
		Cognitive behavioral therapy [28,29,31,46,47]	5	0.35	0.16-0.53	3.64	<.001	0	
	**Number of sessions**								
		< 8 sessions [28,31,46,47]	4	0.37	0.17-0.57	3.63	<.001	0	0.0 (1)
		≥ 8 sessions [29,30]	2	0.34	0.02-0.71	1.83	.07	0	
	**Professional support**									
		Exclusive therapist support [29,31,46,47]	4	0.35	0.16-0.55	3.54	<.001	0	0.1 (1)
		With self-help component [28,30]	2	0.40	0.01-0.80	2.01	.04	0	
**Depressive symptoms [**28-33, 46,47]									
	**Health condition**								
		Depression [28,30-33]	5	0.65	0.48-0.82	7.50	<.001	0	1.8 (2)
		Pregnancy loss [46,47]	2	0.67	0.44-0.90	5.76	<.001	0	
		Post-traumatic stress [29]	1	0.29	0.24-0.82	1.07	.28	N/A	
	**Control condition**								
		Waitlist [29,31,46,47]	4	0.65	0.45-0.85	6.39	<.001	16	0.9 (2)
		Treatment as usual [28,30,32]	3	0.59	0.40-0.78	6.14	<.001	0	
		Waitlist and treatment as usual [33]	1	0.84	0.35-1.33	3.34	<.001	N/A	
	**Type of iCBT**								
		Behavioral activation [30,32]	2	0.56	0.37-0.76	5.62	<.001	0	0. 9 (1)
		Cognitive behavioral therapy [28,29,31,33,46,47]	6	0.69	0.51-0.87	7.62	<.001	0	
	**Number of sessions**								
		< 8 sessions [28,31,46,47]	4	0.73	0.52-0.93	6.96	<.001	0	1.4 (1)
		≥ 8 sessions [29,30,32,33]	4	0.57	0.40-0.74	6.45	<.001	0	
	**Professional support**								
		Exclusive therapist support [29-31,46,47]	5	0.65	0.47-0.84	6.92	<.001	0	0.1 (1)
		With self-help component [28,32,33]	3	0.62	0.43-0.80	6.39	<.001	0	

^a^iCBT, Therapist-supported Internet-based cognitive behavioral therapy.

## Discussion

In this meta-analysis, the efficacy of the therapist-supported iCBT on stress, anxiety, and depressive symptoms during the postpartum period was searched through 10 databases. This study included 8 RCTs involving 1523 postpartum women using iCBT across 6 countries. The results revealed that iCBT significantly improved stress (*d=* 0.84, n=5), anxiety (*d=* 0.36, n=6), and depressive symptoms (*d=* 0.63, n=8) of the intervention group compared with those of the control group at post-intervention.

### Quality of the Evidence and Potential Biases

Data were independently extracted, checked, and entered. The methodological quality of the eligible studies was rated to assess the subjective bias. The overall methodological quality of the studies included in the review was mixed. All studies used methods that we judged to have low risk of bias to randomly assign participants to either the intervention or the control group. This result was due to the selection criteria for RCT. Thus, RCTs prevented selection bias and were insured against accidental bias. All studies achieved adequate allocation concealment. Therefore, participants were unlikely to have selection bias.

A potentially important source of bias in this meta-analysis was that only 12.5% (1/8) of the studies achieved the blinding of participants and personnel. Concealing treatment conditions from participants was impossible because of the control conditions used (eg, waitlist). The results might be influenced by performance bias as concealing of treatment conditions was not possible. Only 12.5% (1/8) of the studies achieved effective blinding of outcome assessment, perhaps primarily owing to the characteristics of the interventions. Hence, a high risk of detection bias remained possible for outcomes relying on self-report or objective outcomes by outcome assessors who were not blinded to treatment allocation. The overall effect of the sample attrition had a low risk of bias in more than half of the studies (62.5%, 5/8), which could improve the generalizability of findings and reduce attrition bias. All studies reported outcomes in a pre-specified manner. Consequently, the eligible studies did not give misleading results because of the selective reporting of outcomes.

In addition, the attrition rates for the intervention and control groups were widely ranged (0%-63.8%). Providing a reminder or tracking system at pre-specified times to intervention users may reduce attrition and ultimately enhance outcomes [29]. Website contact system [29] and phone call [33] may be employed to remind postpartum women to use the site, point to helpful resources, or provide a connection with the treatment team. More than half of the trials (62.5%, 5/8) used ITT analysis, which is an analysis method for solving non-compliance and missing outcomes [56]. Therefore, half of the trials avoided overoptimistic estimates of the efficacy of therapist-supported iCBT by analysing outcomes according to original treatment allocation rather than only for participants completing treatment [56].

### Therapist-Supported Internet-Based Cognitive Behavior Therapy

Consistent with a previous review [25], our finding revealed that interventions initiated during the postpartum period were effective. The majority of therapist-supported iCBT was using cognitive restructuring as an essential element to identify, dispute, and correct irrational or maladaptive thoughts [3]. In our review, therapist-supported iCBT provides a feasible, efficacious, accessible, and economically sound intervention for postpartum women with depressive symptoms [28,30-33], post-traumatic stress symptoms [29], and pregnancy loss [46,47]. Therapist-supported iCBT may be particularly useful for postpartum depressive women who struggle with issues of stigma [30,32]. Therapist-supported iCBT helps women with child-related traumatic event to avoid their fearful impulses, thus helping them to decrease the level of fear experienced at a given moment, and eventually help to confront their fears [29]. In addition, therapist-supported iCBT evidently had great practical significance for women with painful memory of the pregnancy loss in order to improve stress, anxiety, and depressive symptoms [46,47].

Our meta-analytic results found no significant difference on anxiety and depressive symptoms using behavioral activation [4] compared with other forms of CBT. This result suggested that scheduling activities could behaviorally activate women and help them to gain a sense of pleasure and improve anxiety and depressive symptoms [4]. Contrary to previous findings on the equivalent effect of support type for stress symptoms in a meta-analytic review [13], our finding showed that exclusive therapist support has a better effect than therapist support with self-care component in the subgroup analysis. A possible interpretation of this result is that the different attrition rates reached 2.9%–60.8% by therapist support with self-care components [28,32,33] compared with the 7.0%–31.3% rates by exclusive therapist support [29-31,46,47]. This finding supported the fact that exclusive therapist support was preferred to reduce stress symptoms, whether by phone, email, text, or interactive computer interface [10,11]. Our review highlighted that support via website reminder or tracking [28], website contact system [29], or phone calls [33] increased retention to therapist-supported iCBT.

### Efficacy of Therapist-Supported iCBT on Stress Symptoms

The finding of this meta-analysis provided support for the efficacy of therapist-supported iCBT on improving stress symptoms with large effect size of 0.84. This finding is in line with a recent meta-analysis that demonstrated a medium to large effect of 0.72-0.82 of iCBT for post-traumatic stress [13]. Similar components and strategies of iCBT possibly exist among a homogenous dataset with comparable health care conditions in both reviews. However, our result was contradictory with another review [38] indicating inconsistent effects of −0.32 to 0.98. One possible explanation for this difference might be linked to the different target population and health conditions between our review (postpartum women with pregnancy loss, depression, and post-traumatic stress) and those in the previous review (antenatal, postpartum women, and partners with general mental health, grief, stress, post-traumatic stress) [38]. Subgroup analyses revealed a trend for the waitlist comparator to have higher effect sizes than the TAU comparator for improving stress symptoms. The observation echoed a previous review that different control conditions led to substantively different effect estimates and that waitlist comparator generated larger effect sizes estimate than TAU comparator [57]. This finding could be explained by the possibility of additional stress during waiting period because those among waitlist comparator could not access intervention promptly, whereas those assigned to TAU might actively seek additional support that could possibly improve stress symptoms [57].

### Efficacy of Therapist-Supported iCBT on Anxiety Symptoms

Therapist-supported iCBT revealed significantly improved anxiety symptoms with small effect size (*d=* 0.36), which was different from the effect size of the three recent meta-analyses among children (*d=* 0.69) [7], adults with anxiety disorders (*d*=0.79) [10], perinatal women with mixed results indicating both positive effect (*d*=0.51) and negative effect (*d*=−0.61) [38]. The discrepancy might be due to the difference population with specific health conditions between our review (postpartum women) and those in the previous three reviews (childhood and adults with anxiety disorders [7,10] or combination of antenatal, postpartum women, and their partners [38]). Another possible explanation was the substantial heterogeneity in the previous meta-analysis (*I*^2^=80%) [10] compared with homogeneity in our review (*I*^2^=0%). One possible explanation for the differences could be high normative levels of anxiety during the postpartum period [20] because of the conspicuous changes to the roles, lifestyles, and responsibilities of the expecting mother [17]. Directly comparing the current findings was impossible because the evidence was based on different populations with different health condition. This result encourages future investigations.

### Efficacy of Therapist-Supported iCBT on Depressive Symptoms

Therapist-supported iCBT revealed significantly improved depressive symptoms with medium effect size of 0.63 in this review, which was better than the small to medium effect size of 0.41 [36] and 0.56 [37] among adult depression in the other two reviews. This result was consistent with a previous meta-analysis [38] that demonstrated a medium (*d*=0.55) to large (*d*=1.03) effect of intervention for depressive symptoms. The case might be linked to the number of includable trials, types of samples, and variety of therapies in the current review compared with those in the three previous reviews [36-38]. Our review used 8 trials, whereas the previous meta-analytic reviews employed 19 [37] and 12 [36] trials. These two reviews included very heterogeneous therapeutic treatments and samples with substantial to considerable heterogeneity (*I*^2^*=* 57%–81%) [36,37] compared with homogeneity (*I*^2^*=* 0%) in our review. However, similar effect size between one previous meta-analysis [38] and our review might be explained by overlapping with 4 eligible studies [30,32,46,47] in both reviews. Since the previous review [38] did not report results of heterogeneity, it is hard to give a direct comparison. The number of includable trials is relatively few in the current review. Therefore, this aspect should be further explored in future meta-analytic reviews.

The number of includable studies for the subgroup analysis was low in this review, limiting statistical power. The comparisons were unbalanced. We did not find a significant difference between other subgroups, which could be caused by power problems. Further investigations are required.

### Limitations

Several limitations exist in this review. First, a possible sampling bias was evident because the majority of the participants were self-selected on the basis of media-recruited participants rather than clinical samples. Such recruitment methods often rely on the individual’s motivation levels, which potentially correspond to slightly different demographics from those participants who are recruited within community settings. Second, majority of the outcomes were self-reported. Studies typically relied on self-report measures and rarely included formal diagnostic procedures at either recruitment or assessment. As a result, the health conditions or outcomes might be overestimated or underestimated. Third, relatively few trials were included in this review. Therefore, we were underpowered to detect effects for certain contrasts in subgroup analyses. Fourth, different sessions of iCBT tended to report different effect sizes. Fifth, only the short-term benefits of iCBT were investigated on the basis of postintervention outcome measures. The maintenance of benefits in iCBT remains unclear. Wide range of health conditions (especially for stress symptoms, including general and post-traumatic stress), high attrition rate, and broadly defined range of interventions were also considered as limitations of this review. Finally, this review only included studies published in English, and all were conducted in developed regions. The results may not be applicable to marginalized groups in developing regions.

### Implications of Practice

This meta-analytic review extends the evidence of efficacy of iCBT on stress, anxiety, and depressive symptoms among postpartum women. As society becomes increasingly comfortable with, and reliant upon, the use of the Internet for routine health care, opportunities to apply iCBT tend to grow continuously [8]. The iCBT features distinct behavioral advice and learnable skills according to one’s own pace without stigma, waiting times, or taking time off work [16]. Investigating differential predictors of outcomes for another therapeutic format is important. An enhanced understanding of the effective components is necessary to appropriately inform future evidence-based use of iCBT among postpartum women.

### Implications for Future Research

Notably, the evidence of iCBT aims at preventive purpose that employs group-administrated method and exclusive self-help approach among postpartum women are entirely lacking. Future research is welcome. Further investigation is needed on the relationships between the effects of iCBT and different periods of intervention (ie, antenatal vs postpartum), health conditions, and age groups to inform potential iCBT procedures. Participant characteristics may be directly or indirectly associated with attrition rate through a variety of mechanisms, such as literacy, familiarity with technology, personal preferences, and engagement maintenance. This review revealed that different supportive types might affect the different stress symptoms. Ironically, the concept of support is not fully investigated in the literature [36], and we cannot exclude the possibility that some forms of support can be automated. The concepts of support must be further investigated. Although the effects are superior in exclusive therapist support, determining the optimal levels of support is the next issue. The roles of therapist factors, different types of professionals, and support of professionals or non-professionals require further examination. Further rigorous trials based on clinical populations using accurate diagnostic assessment are required before therapist-supported iCBT can be deemed effective for the postpartum population in accessing regular clinical practice for improving stress, anxiety, and depressive symptoms.

### Conclusions

This meta-analytic review supports the efficacy of therapist-supported iCBT for improving stress, anxiety, and depressive symptoms at post-treatment with small to large effects within a range between 0.36 and 0.84. A one-size-fits-all approach is unlikely to succeed considering the complexities and idiosyncrasies of specific health conditions. Future studies should establish the effective components, format, and approach of iCBT with optimal levels of human support. Adequate number of sessions and suitable duration during the postpartum period using appropriate functionality, interactivity, multimedia, and communication modes are important to maximize a long-term effect of iCBT among postpartum women.

## Appendix

Multimedia Appendix 1 Index and keyword terms for searching in ten databases.

Multimedia Appendix 2 Risk of bias graph.

Multimedia Appendix 3 Description of Internet-based cognitive behavioral therapy in 8 trials.

Multimedia Appendix 4 Interaction of Internet-based cognitive behavioral therapy in 8 trials.

## Abbreviations

BSI: Brief Symptom Inventory
CBT: Cognitive Behavior Therapy
DASS: Depression Anxiety Stress Scale
iCBT: Internet-Based Cognitive Behavior Therapy
IES: Impact of Event Scale
IES-R: Impact of Event Scale-revised
ITT: Intention-to-Treat
IV: inverse variance
PICOS: Patient/Problem Intervention Comparison Outcome Setting
PRISMA: Preferred Reporting Items for Systematic Reviews and Meta-analysis
PSS: Perceived Stress Scale
RCT: randomized controlled trial
TAU: treatment as usual
WHO: World Health Organization
