# Supplementary material for: Therapist-Supported Internet-Based Cognitive Behavior Therapy for Stress, Anxiety, and Depressive Symptoms Among Postpartum Women: A Systematic Review and Meta-Analysis
Source: J Med Internet Res. 2017 Apr 28;19(4):e138. doi: 10.2196/jmir.6712 (PMC5429436; doi:10.2196/jmir.6712)
Supplement: Multimedia Appendix 1 [file jmir_v19i4e138_app1.pdf]

Multimedia Appendix 1. Index and keyword terms for searching in ten databases.

Database	Indexed and keyword terms
Academic Search Complete	(((support* OR assist*) W3 (online* OR internet* OR computer* OR 'web based' OR phone OR Skype OR e-therapy OR etherapy) N6 cognitive W3 (behavio?r OR behavioral) W3 (therap* OR treatment* OR intervention* OR management*) OR ccbt OR icbt) OR ((MH "Cognitive Therapy+") AND ((MH "Therapy, Computer Assisted+") OR (MH "Internet+") OR (MH "User-Computer Interface+")))) AND ((antenatal* OR antepartum OR prenatal* OR pre-natal* OR perinatal* OR peripartum* OR puerper* OR pregnan* OR maternal* OR labor*) OR ((MH "Perinatal Care") OR (MH "Pregnancy+")))
CINAHL	(((support* OR assist*) W3 (online* OR internet* OR computer* OR 'web based' OR phone OR Skype OR e-therapy OR etherapy) N6 cognitive W3 (behavio?r OR behavioral) W3 (therap* OR treatment* OR intervention* OR management*) OR ccbt OR icbt) OR ((MH "Cognitive Therapy+") AND ((MH "Therapy, Computer Assisted+") OR (MH "Internet+") OR (MH "User-Computer Interface+")))) AND ((antenatal* OR antepartum OR prenatal* OR pre-natal* OR perinatal* OR peripartum* OR puerper* OR pregnan* OR maternal* OR labor*) OR ((MH "Perinatal Care") OR (MH "Pregnancy+")))
Cochrane	#1 (support* or assist*) and (online* or internet* or computer* or "web-based" or phone or Skype or e-therapy or etherapy) and cognitive and (behavior?r or behavioural) and (therapy or therapies or treatment* or intervention* or management*) or ccbt or icbt:ti,ab,kw<br>#2 MeSH descriptor: [Behavior Therapy] explode all trees<br>#3 MeSH descriptor: [Therapy, Computer-Assisted] explode all trees<br>#4 MeSH descriptor: [Internet] explode all trees<br>#5 MeSH descriptor: [Computers] explode all trees<br>#6 #3 or #4 or #5<br>#7 #2 and #6<br>#8 #1 or #7<br>#9 MeSH descriptor: [Prenatal Care] explode all trees<br>#10 MeSH descriptor: [Perinatal Care] explode all trees<br>#11 MeSH descriptor: [Pregnancy] explode all trees<br>#12 #9 or #10 or #11<br>#13 antenatal* or antepartum or prenatal* or pre-natal* or perinatal* or peripartum* or puerper* or pregnan* or maternal* or labor:ti,ab,kw<br>#14 #12 or #13<br>#15 #8 and #14
Embase	(support* OR assist*) NEXT/3 (online* OR internet* OR computer* OR 'web based' OR phone OR skype OR 'e therapy' OR etherapy) NEAR/6 cognitive NEXT/3 (behavio?r OR behavioral) NEXT/3 (therap* OR treatment* OR intervention* OR management*) OR ccbt OR icbt OR ('cognitive therapy'/exp AND ('computer assisted therapy'/exp OR 'internet'/exp OR 'computer'/exp)) AND (antenatal* OR antepartum OR prenatal* OR 'pre natal*' OR perinatal* OR peripartum* OR puerper* OR pregnan* OR maternal* OR labor* OR ('prenatal care'/exp AND 'perinatal care'/exp) OR 'pregnancy'/exp OR 'maternal care'/exp OR 'puerperal depression'/exp)
ProQuest	(ti(((support* OR assist*) AND (online* OR internet* OR computer* OR 'web based' OR phone OR Skype OR e-therapy OR etherapy) AND cognitive AND (behavio?r OR behavioral) AND (therapy* OR treatment* OR intervention* OR management*) OR ccbt OR icbt)) OR ab(((support* OR assist*) AND (online* OR internet* OR computer* OR 'web based' OR phone OR Skype OR e-therapy OR etherapy) AND cognitive AND (behavio?r OR behavioral) AND (therapy* OR treatment* OR intervention* OR management*) OR ccbt OR icbt)) OR (MESH.EXACT("Behavior Therapy") AND (MESH.EXACT("Therapy, Computer-Assisted") OR MESH.EXACT("Computers") OR MESH.EXACT("Internet")))) AND (ti((antenatal* OR antepartum OR prenatal* OR prenatal* OR perinatal* OR peripartum* OR puerperal* OR pregnan* OR maternal* OR labor*)) OR ab((antenatal* OR antepartum OR prenatal* OR prenatal* OR perinatal* OR peripartum* OR puerperal* OR pregnan* OR maternal* OR labor*)) OR (MESH.EXACT("Prenatal Care") OR MESH.EXACT("Perinatal Care") OR MESH.EXACT("Pregnancy")))

Appendix (*Continued*)

Database	Indexed and keyword terms
PsycArticle	(((support* or assist*) adj3 (online* or internet* or computer* or 'web based' or phone or Skype or e-therapy or etherapy) adj6 cognitive adj3 (behavio?r or behavioral) adj3 (therap* or treatment* or intervention* or management*)) or ccbt or icbt).mp. and (antenatal* or antepartum or prenatal* or pre-natal* or perinatal* or peripartum* or puerper* or pregnan* or maternal* or labor*).mp.
PsycINFO	((((support* or assist*) adj3 (online* or internet* or computer* or 'web based' or phone or Skype or e-therapy or etherapy) adj6 cognitive adj3 (behavio?r or behavioral) adj3 (therap* or treatment* or intervention* or management*)) or ccbt or icbt).mp. OR ((exp cognitive behavior therapy/ or exp Cognitive Therapy/ or exp Behavior Therapy/) and (exp Computer Assisted Therapy/ or exp Computers/ or exp INTERNET/))) AND ((antenatal* or antepartum or prenatal* or pre-natal* or perinatal* or peripartum* or puerper* or pregnan* or maternal* or labor*).mp. or exp Prenatal Care/ or exp Pregnancy/ or exp perinatal period/)
PubMed	(((((support* OR assist*) AND (online* OR internet* OR computer* OR "web-based" OR phone OR Skype OR e-therapy OR etherapy) AND cognitive AND (behavior?r OR behavioural) AND (therapy OR therapies OR treatment* OR intervention* OR management*) OR ccbt OR icbt)) OR ("Behavior Therapy"[Mesh] AND ("Therapy, Computer-Assisted"[Mesh] OR "Internet"[Mesh] OR "Computers"[Mesh])))) AND (((antenatal* OR antepartum OR prenatal* OR pre-natal* OR perinatal* OR peripartum* OR puerper* OR pregnan* OR maternal* OR labor)) OR ("Prenatal Care"[Mesh] OR "Perinatal Care"[Mesh] OR "Pregnancy"[Mesh]))
Scopus	TITLE-ABS-KEY (((support* OR assist*) AND (online* OR internet* OR computer* OR "web-based" OR phone OR Skype OR e-therapy OR etherapy) AND cognitive AND (behavior?r OR behavioural) AND (therapy OR therapies OR treatment* OR intervention* OR management*) OR ccbt OR icbt) AND (antenatal* OR antepartum OR prenatal* OR pre-natal* OR perinatal* OR peripartum* OR puerper* OR pregnan* OR maternal* OR labor))
Web of Science	TOPIC: (((support* OR assist*) AND ((online* OR internet* OR computer* OR 'web based' OR phone OR Skype OR e-therapy OR etherapy) AND cognitive AND (behavio$r OR behavioral) AND (therap* OR treatment* OR intervention* OR management*)) OR ccbt OR icbt) AND (antenatal* OR antepartum OR prenatal* OR pre-natal* OR perinatal* OR peripartum* OR puerper* OR pregnan* OR maternal* OR labor*)) Indexes=SCI-EXPANDED, SSCI, A&HCI, CPCI-S, CPCI-SSH, ESCI Timespan=All years
