# Supplementary material for: Therapist-Supported Internet-Based Cognitive Behavior Therapy for Stress, Anxiety, and Depressive Symptoms Among Postpartum Women: A Systematic Review and Meta-Analysis
Source: J Med Internet Res. 2017 Apr 28;19(4):e138. doi: 10.2196/jmir.6712 (PMC5429436; doi:10.2196/jmir.6712)
Supplement: Multimedia Appendix 2 [file jmir_v19i4e138_app2.pdf]

Multimedia Appendix 2. Risk of bias graph.

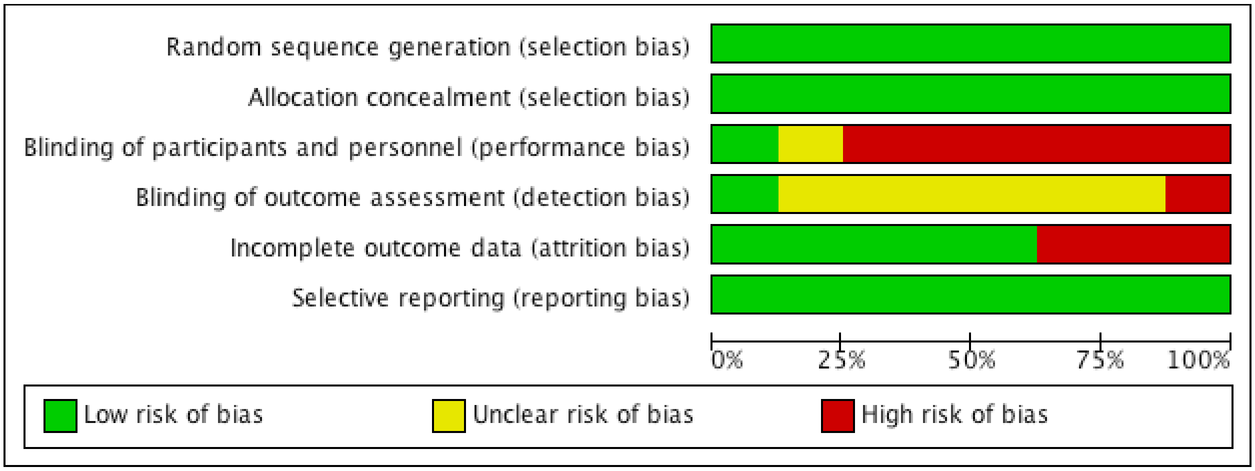

Note: review authors' judgements about each risk of bias item presented as percentages across all included studies.
