# Supplementary material for: Therapist-Supported Internet-Based Cognitive Behavior Therapy for Stress, Anxiety, and Depressive Symptoms Among Postpartum Women: A Systematic Review and Meta-Analysis
Source: J Med Internet Res. 2017 Apr 28;19(4):e138. doi: 10.2196/jmir.6712 (PMC5429436; doi:10.2196/jmir.6712)
Supplement: Multimedia Appendix 3 [file jmir_v19i4e138_app3.pdf]

Multimedia Appendix 3. Description of Internet-based cognitive behavioral therapy in 8 trials

Author (year)	Ref	Aim	No	Components of iCBT Intervention	Therapy	Support	Duration
Kersting et al (2011)	[47]	Treatment	6S	3 phases: self-confrontation, cognitive restructuring and social sharing	One-to-one	Therapist-assisted	5W
Kersting et al (2013)	[46]	Treatment	6S	3 phases: self-confrontation, cognitive reappraisal and social sharing	One-to-one	Therapist-assisted	5W
Milgrom et al (2016)	[28]	Treatment	6S	Getting start, managing mood, increasing pleasant activities and positive though, managing negative thoughts and planning for future.	One-to-one	Therapist-assisted and self-monitoring	6W
Nieminen et al (2016)	[29]	Treatment	8S	Psychoeducation, anxiety coping, method, skill training, imaginary, vivo exposure and cognitive restructuring	One-to-one	Therapist-assisted	8 W
O'Mahen et al (2013)	[32]	Treatment	11S	Behavioural activation including functional analysis, alternative, problem solving, ruminations strategies and relapse prevention	One-to-one	Therapist-assisted and self-monitoring	15W
O'Mahen et al (2014)	[30]	Treatment	12S	Behavioural activation including functional analysis, alternative, problem solving, ruminations strategies and relapse prevention	One-to-one	Therapist-assisted	17W
Pugh et al (2016)	[31]	Treatment	7S	Psychoeducation, activity planning, relaxation, thinking style, cognitive restructuring, problem solving and relapse prevention	One-to-one	Therapist-assisted	10W
Sheeber et al. (2012)	[33]	Treatment	8S	Behavioural activation, interpersonal skills, cognitive skill, managing positive and negative thoughts, planning for future and parenting focus	One-to-one	Therapist-assisted and self-monitoring	14W

Aim, Aim of intervention; iCBT, Internet-based Cognitive Behavioural Therapy; No, Number of session; S, Sessions; W, Week.
