# Supplementary material for: Therapist-Supported Internet-Based Cognitive Behavior Therapy for Stress, Anxiety, and Depressive Symptoms Among Postpartum Women: A Systematic Review and Meta-Analysis
Source: J Med Internet Res. 2017 Apr 28;19(4):e138. doi: 10.2196/jmir.6712 (PMC5429436; doi:10.2196/jmir.6712)
Supplement: Multimedia Appendix 4 [file jmir_v19i4e138_app4.pdf]

Multimedia Appendix 4. Interaction of Internet-based cognitive behavioral therapy in 8 trials.

Author (year)	Ref	Provider (Detail)	Peer	Partner	Contact	Communication /Interactivity	Activities/ Multimedia
Kersting et al (2011)	[47]	CBT trained internet therapist (Psychologists)	No	No	Website	Asynchronous two-way supportive feedback	Text-based Assignment Expressive writing
Kersting et al (2013)	[46]	CBT trained internet therapist (Psychologists)	No	No	Website	Asynchronous two-way supportive feedback	Text-based Assignment Letter writing
Milgrom et al (2016)	[28]	Therapist (Psychology, Clinical psychologist, Health psychologist)	Yes Chat	Yes Partner support website	Phone guided Website reminder or tracking	Synchronous two-way supportive feedback (Telephone coaching)	Text-based Animation Case vignettes Video, audio tutorial Photos Workbook
Nieminen et al (2016)	[29]	Therapist (Psychologist and MSc psychology students)	No	No	Website Contact system (request)	Asynchronous two-way supportive feedback	Text-based Therapy track Assignment track
O'Mahen et al (2013)	[32]	Therapists (Specialist health visitor and clinical psychologist)	Yes Chat	No	Website Phone E-mail	Asynchronous two-way supportive feedback	Multimedia Online clinic Assignment
O'Mahen et al (2014)	[30]	Therapist (Mental health workers)	Yes Chat	No	Website E-mail Phone Text messages	Asynchronous two-way supportive feedback	Interactive exercise with work examples Audio
Pugh et al (2016)	[31]	Therapist (PhD clinical psychology students and psychologist)	No	No	Email Phone	Asynchronous two-way supportive feedback	Assignment Text /Animation Video / Audio Check-in questions
Sheeber et al. (2012)	[33]	CBT trained therapist (Mental health worker and lay person)	Yes Chat	No	Website Phone	Asynchronous two-way supportive feedback	Board writing Bulletin board Online consultation

Chat, Chat Room Discussion; Contact, Contact with therapist; Partner, Partner Support; Peer, Peer Support
